# Reply to Kiral, B.S. Ultrasonographic Evaluation of Superior Cluneal Nerve Entrapment: Methodological Considerations. Comment on “Iudicelli et al. The Role of Musculoskeletal Ultrasound in Detecting Superior Cluneal Nerve Entrapment: Biomechanical Insights in Chronic Low Back Pain—A Pilot Study. *Diagnostics* 2026, *16*, 469”

**DOI:** 10.3390/diagnostics16152472

**Published:** 2026-08-05

**Authors:** Giovanni Iudicelli, Francesco Agostini, Alberto Altarocca, Francesco Ioppolo, Marco Narciso, Marco Conti, Andrea Fisicaro, Alessio Savina, Vincenzo Di Nunno, Massimiliano Mangone, Stefano Galletti, Marco Paoloni

**Affiliations:** 1 Department of Anatomy, Histology, Forensic Medicine and Orthopedics, Sapienza University, 00185 Rome, Italy; francesco.agostini@uniroma1.it (F.A.); marco.narciso@uniroma1.it (M.N.); ma.conti@uniroma1.it (M.C.); andrea.fisicaro@uniroma1.it (A.F.); alessio.savina@uniroma1.it (A.S.); massimiliano.mangone@uniroma1.it (M.M.); marco.paoloni@uniroma1.it (M.P.); 2 Rehabilitation Unit, University Hospital Umberto I, 00185 Rome, Italy; a.altarocca@policlinicoumberto1.it (A.A.); f.ioppolo@policlinicoumberto1.it (F.I.); 3 “Azienda ULSS 9 Scaligera”, 37122 Verona, Italy; vincenzo.dinunno@aulss9.veneto.it; 4 Musculoskeletal Ultrasound School, Italian Society for Ultrasound in Medicine and Biology, 40124 Bologna, Italy; stefanogalletti2011@gmail.com

## Abstract

We thank Dr. Kiral for a thoughtful and constructive commentary, with which we largely agree. Surrogate biomechanical markers and direct visualisation of the SCN are complementary rather than competing: the markers were intended to extend diagnostic support to the many settings and patients in whom direct visualisation is not easy to achieve, whether for patient-related reasons or because high-resolution equipment and dedicated expertise are not universally available. We agree that these findings are supportive rather than primary diagnostic markers, and that their value may lie in the triad within a clinically pre-selected population rather than in any single sign. The nociceptive–neuropathic distinction the author emphasises is itself one of the central conclusions of our study.

## 1. Accessibility: The Rationale for Surrogate Markers

We appreciate Dr. Kiral’s careful engagement [1] with our pilot study. We agree that direct sonographic visualisation of the SCN is achievable with high-resolution equipment in experienced hands [2], as we acknowledged in our article. Our argument concerns accessibility and reproducibility rather than feasibility in principle. Two constraints limit direct visualisation in practice. The first is patient-related: with a mean calibre of approximately 1.1 mm and a variable course, the SCN is difficult to identify confidently when elevated body mass, subcutaneous adiposity, or unfavourable tissue echogenicity interfere, factors we noted in our article [3] and that operate regardless of operator skill. The second is technical: as noted by Kiral, direct SCN depiction is more likely when high-resolution ultrasound is available and appropriate scanning technique and operator experience are ensured [1]. Such resources are not uniformly available across the settings in which chronic low back pain is actually seen.

Surrogate biomechanical markers may therefore extend diagnostic support to operators, centres, and patients for whom direct visualisation is not readily achievable, without presupposing the equipment or expertise it demands. Our study did not, however, compare the two approaches in terms of reproducibility or operator dependence; these are offered as potential practical advantages requiring formal comparison. The two approaches answer different questions. Direct visualisation confirms the course and location of the nerve but does not by itself establish entrapment, which remains a clinical diagnosis; the markers characterise the biomechanical environment that may predispose to it. We fully share the author’s position that these findings are supportive rather than primary, a restatement of our own design, since we used ultrasound not for primary diagnosis, which rests on the clinical picture, but to investigate surrogate markers within an already clinically identified population [3].

## 2. The Triad and the Role of Clinical Context

We agree that individual markers such as thoracolumbar fascia (TLF) thickening or fibro-adipose nodules are not, in isolation, specific to SCN entrapment and may occur in other forms of chronic low back pain; this is precisely why our proposal concerns a triad, rather than any single sign. By “triad,” we mean the three markers interpreted together as a composite biomechanical pattern, and not a diagnostic rule requiring all three findings to be present in every patient. We propose that its diagnostic value may arise from the conjunction of the three markers in a population already pre-selected on the SCN distribution, in which facet arthropathy, sacroiliac dysfunction, and active radiculopathy had been systematically excluded [3]. In that context, iliac enthesophytes were present in 58.3% of patients but in none of the controls (*p* = 0.005) [3]. We should emphasise, however, that the combined performance of the three markers was not formally evaluated: no sensitivity, specificity, or predictive values were computed for the pattern, and no composite index was derived or internally validated. Each marker considered in isolation is non-specific, and we can therefore propose only that their joint occurrence within an appropriate clinical context may plausibly enhance specificity; this remains a hypothesis requiring formal testing.

## 3. Copeman Nodules

We noted that Copeman nodules are often asymptomatic and treated them as one associative component of the triad, not a stand-alone marker [3]. We also agree that their nature should be reconsidered: their fibro-adipose character and frequent location beneath the superficial fascia without clear herniation [4,5] challenge the traditional definition, in keeping with the historical reappraisal of sacroiliac fatty nodules [6]. Notably, in our cohort, every nodule lay beneath the superficial fascia without herniation [3], converging directly with the author’s observation. No histological confirmation was obtained in our cohort: the term “fibro-adipose” describes their sonographic appearance, in keeping with established terminology, rather than a tissue-level characterisation.

## 4. The Nociceptive–Neuropathic Distinction Is a Shared Conclusion

We welcome the author’s emphasis on distinguishing fascia-related nociceptive pain from neuropathic pain secondary to SCN entrapment, since this is one of the principal conclusions of our study. The TLF is richly innervated by nociceptive fibres, likely Aδ and C type [7], and our data show the same dissociation: TLF thickness correlated strongly with the Numeric Pain Rating Scale (ρ = 0.825, *p* = 0.001) but not with the DN4 neuropathic score [3], which may be consistent with a predominantly fascial nociceptive mechanism rather than direct nerve damage.

## 5. Concluding Remarks

Direct visualisation and surrogate markers are complementary: the former is invaluable where achievable, while the latter may provide additional support where direct visualisation is not readily achievable. The strength of the markers may lie in their combination within a clinically defined population, and the nociceptive–neuropathic distinction is one we share. We agree that the field now requires multicentre prospective studies with larger cohorts and standardised ultrasonographic criteria, and we thank the author for advancing this discussion.

## Data Availability

No new data were created or analysed in this study. Data sharing is not applicable.
